# Health Care Professionals’ Evaluation of Quality of Life Issues in Patients With Brain Metastases

**DOI:** 10.4021/wjon584w

**Published:** 2013-01-04

**Authors:** Natalie Lauzon, Gillian Bedard, Liying Zhang, Arjun Sahgal, Liang Zeng, Kaitlin Koo, Edward Chow

**Affiliations:** a Rapid Response Radiotherapy Program, Department of Radiation Oncology, Odette Cancer Centre, Sunnybrook Health Sciences Centre, University of Toronto, Toronto, Ontario, Canada

**Keywords:** Brain metastases, Health care professionals, Oncology, FACT-Br

## Abstract

**Background:**

The Functional Assessment of Cancer Therapy - Brain (FACT-Br) is a brain specific Quality of life (QOL) tool used for patients in the primary and metastatic cancer population. The purpose of this report is to evaluate the QOL issues health care professionals (HCPs) find most important when caring for brain metastases patients.

**Methods:**

HCPs were asked to rate whether each of the 23 FACT-Br subscale items were relevant to patients or not. In the survey, HCPs indicated the 5 to 10 top issues affecting the QOL of patients with brain metastases. Demographic information such as gender, years of experience, and health care specialty were recorded.

**Results:**

A total of 46 HCPs participated in the study, 89% of HCPs ranked the need for help in caring for themselves as the most relevant item for patients with brain metastases. Other highly relevant items included the concern of getting headaches (81%) and weakness in arms or legs (78%). The lowest rated items included the ability to put thoughts together (8%), ability to write as they used to (11%) and also the ability to read as they used to (14%).

**Conclusion:**

It is very important to determine the issues that HCPs think are most important to patients in an attempt to harmonize these with those of patients. Future studies should compare the items that HCPs rate as most relevant to those that patients rate to ensure agreeability.

## Introduction

Assessments of the patient perspective of quality of life (QOL) play an increasingly important role in clinical oncology, where the primary aim is to better understand the impact of cancer and its treatment [1, 2]. The concept of QOL refers to the physical, psychological and social domains of health, identified as distinct areas that are influenced by an individual’s experiences, perceptions, beliefs and expectations [3]. This multi-dimensional construct has been under development for over 20 years and as of yet, still lacks an international consensus about its measure [4]. In the palliative care setting, there is increasing recognition that QOL is an outcome that is as important, if not more important, than traditional outcomes such as survival [5, 6]. Major clinical trials in Europe and the United States now advocate the use of QOL assessments and use them as end points [5, 7, 8]. The National Cancer Institute of Canada has also mandated organizational requirements for inclusion of QOL endpoints in clinical trials thus potentially providing an increased exposure and use of QOL assessments by clinicians [9].

With current emphasis on QOL, it is important that both patients and their health care providers (HCP) not only perceive value in its application, but share agreement in the issues. This is of paramount importance with brain metastases patients and their HCPs given the overall poor prognosis of this patient population.

Brain metastases patients have very discrete symptoms and emotional issues to contend with. Functional impairments resultant from brain metastasis cause considerable distress in cancer patients and their caregivers. The required adaptation to changing functional and social limitations, confusion from the disease itself, along with the multiple treatment decisions all confound the issues that brain metastases patients must encounter. QOL should be achieved by minimizing risks and maximizing benefits of treatment all while including the psychosocial as well as physical elements [10]. An individual's unique meaning of QOL can have great implications for HCPs trying to evaluate medical treatments and QOL interventions. There are many QOL assessment techniques and questionnaires available for use. One such QOL measure that was developed specifically for the brain tumor population is the Functional Assessment of Cancer Therapy-Brain (FACT-BR) [11, 12]. The FACT-Br and its subscale offers a comprehensive look at the emotional, social, psychological, and cognitive aspects of a patient's life and has been validated in the brain tumor population.

In order to obtain a comprehensive understanding of a patient’s physical and psychosocial health status, HCPs must communicate effectively with patients and their families so as to allow a complete understanding of not only the physical but also the psychosocial health status of the patient [13]. The purpose of this report is to evaluate the QOL issues HCPs find most important when caring for brain metastases patients.

## Methods

### Questionnaire

The FACT-Br is a validated, QOL specific assessment tool originally developed for patients with primary brain neoplasms. It consists of 27 items pertinent to all cancer patients (FACT-G) spanning physical, social, emotional and functional QOL domains along with 23 additional items assessing brain specific QOL issues (FACT-Br subscale) [12].

### Health care professionals

HCPs directly involved in the care of patients with brain metastasis were eligible for inclusion in the study. They were asked to rate whether each of the 23 FACT-Br subscale items were relevant to patients or not. In the survey, HCPs indicated the 5 to 10 top issues affecting the quality of life of patients with brain metastases. Demographic information such as gender, years of experience, and health care specialty were recorded. All research conducted in this study was reviewed and approved by the Sunnybrook Health Sciences Centre research ethics board.

### Statistical analysis

Demographic information was summarized as mean, standard deviation (SD), median, and range for years of experience, and as proportions for gender and profession. The number of HCPs’ responses and percentages were calculated to describe the relevance as well as the top 10 relevant items from HCPs. All analyses were conducted by Statistical Analysis Software (SAS version 9.2 for Windows).

## Results

A total of 46 HCPs participated in this study. The median years of professional experience was 5.5 with a relatively equal distribution of male (42%) and female (58%) respondents. The majority of respondents were radiation oncologists (41%) while radiation therapists and nurses made up 17% and 15% respectively (Table 1). When we restricted HCPs who completed relevancy of each item, the male to female ratio of respondents was 47 to 53%, with a median of 5 years of professional experience among 37 HCPs (Table 2).

**Table 1:** Demographics of HCPs

Years of professional experience		
n	46	
Mean ± SD	7.2 ± 6.5	
Inter-quartiles	3 - 10	
Median (range)	5.5 (1 - 25)	
Gender		
Female	26	(57.78%)
Male	19	(42.22%)
Profession		
Radiation Oncologist	19	(41.30%)
Radiation Therapist	8	(17.39%)
Nurse	7	(15.22%)
Medical Oncologist/Haematologist	3	(6.52%)
Palliative Care Physician	2	(4.35%)
General Practitioner in Oncology	1	(2.17%)
Neurosurgeon	1	(2.17%)
Others	5	(10.87%)

**Table 2:** Demographics of HCPs Who Completed Relevancy of Each Item

Years of professional experience		
n	37	
Mean ± SD	6.8 ± 6.6	
Inter-quartiles	2 - 10	
Median (range)	5.0 (1 - 25)	
Gender		
Female	19	(52.78%)
Male	17	(47.22%)
Profession		
Radiation Oncologist	19	(51.35%)
Nurse	6	(16.22%)
Radiation Therapist	3	(8.11%)
Medical Oncologist/Haematologist	2	(5.41%)
General Practitioner in Oncology	1	(2.70%)
Neurosurgeon	1	(2.70%)
Palliative Care Physician	1	(2.70%)
Others	4	(10.81%)

Among the total 46 HCPs, there were 37 HCPs with available responses on Relevant of QOL.

Of the 46 HCPs in this survey, there were 39 available respondents who completed the relevance rating of yes or no to each of the 23 FACT-Br subscale items. All items with the exception of 6 were rated as relevant by greater than 50% of HCPs. Items that were less relevant included: being afraid of having a seizure (convulsions) (41%), being bothered by the change in personality (49%) as well as drop in contribution to the family (46%), ability to put thoughts together (41%) as well as into action (44%) and the ability to write like they used to (44%). Items such as actually having had a seizure or convulsions (87%), having difficulty expressing one’s thoughts (82%), needing help in caring for myself (bathing, dressing, eating) (95%), having weakness in arms or legs (85%) and getting headaches (85%) were all rated as relevant by 80% or more of HCPs (Table 3).

**Table 3:** HCPs’ Responses

	Relevant to Patients		Total
	No n (%)	Yes n (%)	
Additional Concerns			
Br1	11 (28.21%)	28 (71.79%)	39
Br2	5 (12.82%)	34 (87.18%)	39
Br3	19 (48.72%)	20 (51.28%)	39
Br4	19 (48.72%)	20 (51.28%)	39
Br5	23 (58.97%)	16 (41.03%)	39
Br6	11 (28.21%)	28 (71.79%)	39
Br7	14 (35.90%)	25 (64.10%)	39
NTX6	17 (43.59%)	22 (56.41%)	39
Br8	17 (43.59%)	22 (56.41%)	39
Br9	7 (17.95%)	32 (82.05%)	39
Br10	20 (51.28%)	19 (48.72%)	39
Br11	13 (33.33%)	26 (66.67%)	39
Br12	21 (53.85%)	18 (46.15%)	39
Br13	23 (58.97%)	16 (41.03%)	39
Br14	2 (5.13%)	37 (94.87%)	39
Br15	22 (56.41%)	17 (43.59%)	39
Br16	19 (48.72%)	20 (51.28%)	39
Br17	22 (56.41%)	17 (43.59%)	39
Br18	14 (35.90%)	25 (64.10%)	39
Br19	11 (28.21%)	28 (71.79%)	39
Br20	6 (15.38%)	33 (84.62%)	39
Br21	8 (20.51%)	31 (79.49%)	39
An10	6 (15.38%)	33 (84.62%)	39

39 of 46 available for Use.

In the ranking of top 10 items of relevance for brain metastases patients, there were 37 of the 46 available responses available for frequency analysis, 11 of the 23 FACT-Br items were determined by 50% or more of HCPs to be relevant. 32 of the 39 HCPs (89%) ranked the need for help in caring for themselves (bathing, dressing, eating, etc.) as the most relevant item for patients with brain metastases. This was closely followed (81%) by the concern of getting headaches and weakness in arms or legs (78%). The 3 items of having had seizures (convulsions), having trouble with coordination and having trouble with eyesight were ranked by 24 of the 39 HCPs (67%) as relevant. Having trouble feeling sensations in arms, hands or legs, ability to concentrate and trouble with eyesight were ranked as relevant by 56, 53 and 53% of HCPs respectively. The ability to make decisions and take responsibility, along with feeling independent was deemed relevant by 50% of HCPs. Rounding out the bottom items, deemed relevant by the fewest percentage of HCPs, were items such as the ability to put thoughts together (8%), ability to write as they used to (11%) and also the ability to read as they used to (14%). Table 4 summarizes the top 10 relevant items as assigned by HCPs for brain metastases patients. Table 5 provides a description of the FACT-Br subscale items.

**Table 4:** Percentage of Participants Selecting Each Item as One of Their 10 Most Relevant Items

Item	n	%
Br14	32	88.89%
An10	29	80.56%
Br20	28	77.78%
Br2	24	66.67%
Br21	24	66.67%
Br9	24	66.67%
Br19	20	55.56%
Br1	19	52.78%
Br6	19	52.78%
Br11	18	50.00%
Br7	18	50.00%
Br4	15	41.67%
Br8	14	38.89%
Br18	13	36.11%
Br10	11	30.56%
Br12	10	27.78%
Br3	10	27.78%
NTX6	8	22.22%
Br5	7	19.44%
Br15	6	16.67%
Br16	5	13.89%
Br17	4	11.11%
Br13	3	8.33%

**Table 5:** FACT-Br Subscale

	Additional Concerns	Not at all	A little bit	Some-what	Quite a bit	Very much
Br1	I am able to concentrate	0	1	2	3	4
Br2	I have had seizures (convulsions)	0	1	2	3	4
Br3	I can remember new things	0	1	2	3	4
Br4	I get frustrated that I cannot do things I used to	0	1	2	3	4
Br5	I am afraid of having a seizure (convulsion)	0	1	2	3	4
Br6	I have trouble with my eyesight	0	1	2	3	4
Br7	I feel independent	0	1	2	3	4
NTX6	I have trouble hearing	0	1	2	3	4
Br8	I am able to find the right word(s) to say what I mean	0	1	2	3	4
Br9	I have difficulty expressing my thoughts	0	1	2	3	4
Br10	I am bothered by the change in my personality	0	1	2	3	4
Br11	I am able to make decisions and take responsibility	0	1	2	3	4
Br12	I am bothered by the drop in my contribution to the family	0	1	2	3	4
Br13	I am able to put my thoughts together	0	1	2	3	4
Br14	I need help in caring for myself (bathing, dressing, eating, etc.)	0	1	2	3	4
Br15	I am able to put my thoughts into action	0	1	2	3	4
Br16	I am able to read like I used to	0	1	2	3	4
Br17	I am able to write like I used to	0	1	2	3	4
Br18	I am able to drive a vehicle (my car, truck, etc.)	0	1	2	3	4
Br19	I have trouble feeling sensations in my arms, hands, or legs	0	1	2	3	4
Br20	I have weakness in my arms or legs	0	1	2	3	4
Br21	I have trouble with coordination	0	1	2	3	4
An10	I get headaches	0	1	2	3	4

## Discussion

This report demonstrates that HCPs tend to assign relevance more frequently to issues concerning physical manifestations for patients, rather than psychosocial ones. There appears to be a trend with physical findings such as dressing, bathing and eating being assigned most frequently as relevant, rather than issues involving social and cognitive function such as reading, writing and comprehension.

The top 3 items most commonly rated as relevant by HCPs were needing help in caring for oneself, followed closely by getting headaches and then by having weakness in the arms or legs. Perhaps one of the reasons that the majority of HCPs ranked these items as relevant was that physicians may feel a greater sense of ability to help alleviate physical discomforts and limitations rather than psychosocial ones. QOL issues that are considered less observable such as social functioning are often left unaddressed by health care practitioners [14, 15]. Detmar et al provided results indicating that all oncologists surveyed considered it their responsibility to discuss the physical aspects of patients’ health, yet discussing psychosocial concerns was deemed a venture to be shared among all health care providers. All of these oncologists indicated that these issues are to be initiated for discussion by patients [16]. This pattern appears similar with the ranking of relevance for issues as discussed in this report. When considering the treatment management options for brain metastases patients, HCPs must consider not only the physical aspects but also the psychosocial elements when communicating with patients. In this report, the lowest ranked relevant items were the ability to read, write and put thoughts into action. Levinson and Roter explain that HCPs vary widely in their interest and ability to extract relevant information from their patients and only when physicians had a positive attitude towards the psychosocial aspects of care did the patients disclose significantly more information about their emotional and social functioning [17]. A randomized controlled trial of QOL Assessments and Patient-Physician Communication by Detmar and colleagues concluded that incorporating standardized QOL assessments in daily clinical oncology practice facilitates the discussion of QOL issues and can heighten physicians’ awareness of their patients’ QOL [18].

A study by Bezjak et al reported that 82% of oncologists surveyed expressed that their knowledge about QOL was limited and that they believed in the benefit of QOL assessments for patient care and stated that they would increase their use of QOL information in the future [19]. This further emphasizes the importance of providing an evaluation of QOL issues that matter most to HCP caring for brain metastases patient.

Bezjak and colleagues suggest that there is a discrepancy between international recommendations and the expert opinion that symptoms and QOL domains should be included in the treatment decision and integration into daily clinical practice [20]. Establishing a comprehensive evaluation of QOL issues as deemed relevant to brain metastases patients by their health care providers is an important step in leading towards the regular implementation of HRQOL domains in clinical practice.

The entire realm of QOL must remain the focus of care as this has been shown repeatedly in studies as having an enormous impact on patient satisfaction. Harris’ study compareing the patients’ and HCPs’ evaluation of health related quality of life issues in bone metastases clearly shows that patients’ perception of their QOL encompasses not only the physical construct but also the psychosocial elements [21]. With QOL being a multidimensional construct encompassing not only the physical, psychological and social functioning as well as the disease and treatment related symptoms, HCPs must consider all elements. Brain metastases patients are prone to sudden deterioration in neuro-cognitive function and performance status. As such, it is imperative that patients and their HCPs consider similar issues early on in the management of care process.

This study is not without limitations, including the heterogeneity of the HCPs surveyed. The majority of participants were radiation oncologists (51%) only then followed by 16% of respondents being nurses and 8% radiation therapists. This study was also limited in providing only information based on the issues that are deemed important by HCPs and are not directly comparing to brain metastases patients’ QOL concerns.

When it comes to content in QOL assessment tools, it is generally accepted that the patient perspective is the gold standard [22]. Since patient experiences can be subjective, one’s definition and measure of issues can differ starkly from another. Future studies should explore the similarities between patient and HCP determined QOL item relevance.
